# An Exploration of Sedentary Behavior, Physical Activity, and Quality of Life During the COVID-19 Outbreak

**DOI:** 10.3389/ijph.2023.1605585

**Published:** 2023-01-26

**Authors:** Cheng-Yen Huang, Wen-Hsin Huang, Hsin-Yen Yen

**Affiliations:** ^1^ Changhua Christian Hospital, Changhua, Taiwan; ^2^ Humboldt Park Health, Chicago, IL, United States; ^3^ School of Gerontology and Long-Term Care, College of Nursing, Taipei Medical University, Taipei, Taiwan

**Keywords:** health promotion, active lifestyle, immune status, precautionary behavior, health belief model

## Abstract

**Objectives:** Staying physically active is a cost-efficient strategy for disease prevention during a pandemic. The purposes of this study were to explore precautionary behaviors, psychological factors associated with physical activity and sedentary behavior, and impacts of active and sedentary lifestyles on the quality of life in the early stage of the coronavirus disease 2019 (COVID-19) outbreak.

**Methods:** Participants were community-dwelling adults aged over 20 years who had not been infected with COVID-19 and who lived in the United States. A study with a cross-sectional design was conducted between July and October 2020. Quantitative data were collected by a self-reported questionnaire.

**Results:** In total, 467 valid responses were obtained. Participants who engaged in an active lifestyle had significantly higher scores on all domains of quality of life compared to those who engaged in an inactive lifestyle. Participants with a non-sedentary lifestyle had significantly higher scores of psychological and social domains of quality of life than those with a sedentary lifestyle.

**Conclusion:** Engaging in an active lifestyle and avoiding a sedentary lifestyle are recommended when facing future, unpredictable pandemics similar to COVID-19.

## Introduction

Engaging in physical activity (PA) is an efficient strategy to prevent chronic and infectious diseases in all age groups [1]. Increasing PA and decreasing sedentary behavior (SB) can promote appropriate personal health benefits, strengthen one’s immune system, decrease stress and the threat of infectious diseases, and improve one’s quality of life (QOL), which might have beneficial effects when facing a pandemic [2–5]. However, from a psychological perspective, even if individuals had wanted to go out and participate in PA, they may have been fearful of contacting potentially infected people and sources of infection in the early stage of the coronavirus disease 2019 (COVID-19) pandemic [3].

This pandemic may have changed individuals’ healthy behaviors and lifestyles, especially those who live in crowded cities [5]. Individuals may spend a lot of time engaged in SB of less than or equal to 1.5 metabolic equivalents (MET) during the pandemic, such as just sitting, lying down, watching TV, looking at a computer screen, playing with their smartphones and video games, studying, and reading, instead of participating in PA [6]. Sedentary and inactive lifestyles might lead to further health problems, such as increased severity of symptoms of individuals suffering from COVID-19, reductions in respiratory muscle functions, immune system functions, and other physical health functions, and increases in inflammation, stress, anxiety, depression, and other mental health problems [7–9].

The Health Belief Model (HBM) is a well-known social cognitive model for understanding and explaining PA and SB. The six constructs provide a conceptual framework for both short- and long-term healthy behaviors [10–12]. However, because of quarantine policies in the early stage of the outbreak, individuals’ health beliefs might have changed. Individuals who lacked spaces for PA, changed their former exercise routines, and experienced increased barriers to PA may have had reduced motivation, self-efficacy, cues to action, and health awareness of PA [13, 14]. An individual’s long-time SB increases the risk of, the severity of, and susceptibility to both chronic and infectious diseases [11, 12].

The World Health Organization (WHO) recommends that adults aged 18–64 years should engage in at least 150 min of moderate-intensity aerobic physical activity, at least 75 min of vigorous-intensity aerobic physical activity, or an equivalent combination of moderate- and vigorous-intensity activity throughout the week [1]. Furthermore, outdoor PA with appropriate precautions is still advocated for individuals during a pandemic. The US Centers for Disease Control (CDC) proposed several precautionary approaches when engaging in PA during the COVID-19 outbreak, such as tips for engaging in PA indoors, staying active close to home, doing PA while socially distancing, protecting oneself and others when playing sports, visiting beaches and pools, and visiting parks and recreational facilities [15].

This study focused on examining PA and SB in the early stage of the COVID-19 outbreak. The purposes were to explore precautionary behaviors during outdoor PA, psychological factors associated with PA and SB, and the influences of active and sedentary lifestyles on participants’ QOL. The HBM was applied as psychological factors. The study is expected to assist in facing new and similar pandemics in the future. The research questions were as follows.

1. Does a participant’s demographic background make a difference in precautions taken when engaging in outdoor PA?
2. Were one’s perceived immune status and health beliefs associated with the odds of engaging in an active lifestyle or a sedentary lifestyle?
3. Do participants with an active lifestyle and those with an inactive lifestyle exhibit significant differences in QOL?
4. Do participants with a sedentary lifestyle and those with a non-sedentary lifestyle exhibit significant differences in QOL?

## Methods

### Study Design

This study had a cross-sectional design. An anonymous survey was conducted *via* Amazon Mechanical Turk (MTurk) between July and October 2020. MTurk is a mass-marketing survey tool that provides the largest network platform with a feedback mechanism. It uses crowd-sourcing to obtain resources, and can conduct data verification, investigations, and content review, and accomplish other academic research tasks, to improve the validity and quality of self-reported data [16, 17]. The potential participant pool could stay demographically stable and consistent before and after the pandemic [18]. During the pandemic, quarantine had the potential benefit of making the sample more representative [19].

### Participants

Participants were community-dwelling adults aged over 20 years, who lived in the United States, with no disability, and who had not been infected with COVID-19. Participants who achieved the weekly PA recommendations of the WHO [1] were categorized into the group with an active lifestyle, while those who did not achieve the PA recommendations were categorized into the group with an inactive lifestyle. According to a cutoff of daily sedentary time [20], a participant whose daily sedentary time exceeded 9 h was categorized into the group of sedentary lifestyles, while those with <9 h were categorized into the group of non-sedentary lifestyles. The minimum required sample size in epidemiological cross-sectional studies is 384, which was calculated by an unknown population with a 5% acceptable margin of error and a 95% confidence level [21].

### Data Collection

A self-reported questionnaire was used for data collection. The questionnaire consisted of five parts. Demographic variables included living region, ethnicity, age, gender, educational attainment, marital status, employment status, annual income, and chronic diseases. Two self-reported items asked whether they had any disabilities and had been infected with COVID-19.

### Measurements

#### Precautions Taken During Outdoor PA

In May 2020, the US CDC proposed four things each that people should and should not do when visiting outdoor parks and recreational facilities. People were allowed to [1] visit parks that are close to their home [2]; prepare before they visit [3]; stay at least 6 feet (1.8 m) away from others (“social distancing”) and take other steps to prevent COVID-19; and [4] play it safe around and in swimming pools and keep space between themselves and others. In contrast, people should not [1] visit parks if they are sick or have recently been exposed to COVID-19 [2]; visit crowded parks [3]; use playgrounds; and [4] participate in organized activities or sports [22]. According to these precautions, our questionnaire was designed with eight items with a frequency which was evaluated on a 4-point scale. The score of four items of things that should not be done were reversed to be added to the four items of things that should be done. A higher score represents a higher frequency of precautions taken when engaging in outdoor PA. Cronbach’s ɑ was 0.800.

#### Perceived Immune Status

The Immune Status Questionnaire (ISQ) was used to measure the perceived immune status in the past 6 months. The self-reported immune status is associated with biological immune signs [23]. In total, seven questions of symptoms were measured by a 5-point frequency. The raw score was transformed into a 10-point final scale. A higher final score indicates a better perceived immune status. A cutoff of six points of the final scale was used to divide participants into high and low immune statuses. The ISQ can be used for rapid screening in clinical practice. Individuals with a low immune status are recommended for further medical examination. Cronbach’s ɑ was 0.952.

#### Health Beliefs in PA

A questionnaire was self-developed in accordance with the HBM with good construct validity that was tested by a confirmatory factor analysis and item analysis [14]. In total, 30 questions assessed agreement with a 5-point scale. The six domains were perceived barriers to PA, perceived benefits of PA, social support, cues to action, susceptibility to diseases, and self-efficacy. Cronbach’s ɑ of the various domains ranged 0.716–0.901.

#### PA and SB

The International Physical Activity Questionnaire Short-form (IPAQ-SF) was used to assess participants’ PA and SB in the past week [24]. Participants were asked about seven items of the weekly frequency and the daily minutes of each PA intensity, including walking (light-intensity PA), moderate-intensity PA, vigorous-intensity PA, and sedentary time. The number was multiplied to calculate the weekly minutes of PA and daily minutes of SB.

#### QOL

The World Health Organization Quality-of-Life Scale (WHOQOL-BREF) was used to assess participants’ QOL [25]. In total, 26 items were evaluated on a 5-point scale. The four domains of QOL were physical health, psychological health, social relations, and environment. Cronbach’s ɑ was 0.930.

### Statistical Analysis

Descriptive analyses used frequencies for categorical variables and means and standard deviations (SDs) for continuous variables. Independent *t*-tests and analyses of variance (ANOVAs) with Scheffé's *post hoc* test were applied to analyze differences in precautions taken during outdoor PA among categorical demographic variables. Pearson’s correlation analysis was used to examine associations between age and precautions taken during outdoor PA. A logistical regression was used to examine associations of factors for an active lifestyle and sedentary lifestyle, including one’s perceived immune status and health beliefs in PA. Logistical regression models were adjusted for continuous demographic variables as covariables. Independent *t*-tests were also used to analyze differences in the four QOL domains between active and inactive lifestyles and those with sedentary and non-sedentary lifestyles. SPSS software was used for all statistical analyses.

## Results

### Participant Characteristics

In total, 467 participants validly responded after excluding those who did not meet the inclusion criteria. Participants’ characteristics are shown in Table 1. Participants’ average age was 39.87 ± 12.13 years.

**TABLE 1:** Participants’ characteristics (United States, 2020).

Characteristic		Mean	SD
Age (years)		39.87	12.13
		*n*	(%)
Gender	Male	265	56.75
	Female	202	43.25
Highest education	(1) Less than high school	73	15.63
	(2) Associate or college degree	332	71.09
	(3) Graduate degree	62	13.28
Annual income (US$)	(1) <30,000	166	35.55
	(2) 30,001–50,000	127	27.19
	(3) 50,001–70,000	102	21.84
	(4) >70,001	72	15.42
Ethnicity	(1) White	283	60.60
	(2) Asian	91	19.49
	(3) African-American	63	13.49
	(4) Other	30	6.42
Marital status	(1) Single	145	31.05
	(2) Married	293	62.74
	(3) Other	29	6.21
Employment status	(1) Full-time job	359	76.87
	(2) Part-time job	57	12.21
	(3) Unemployed	24	5.14
	(4) Other	27	5.78
Living environment	(1) Urban area	235	50.32
	(2) Suburban area	166	35.55
	(3) Rural area	66	14.13
Chronic diseases	No	254	54.39
	Yes	213	45.61

SD, standard deviation.

### Demographic Variables and Precautions Taken During Outdoor PA

Differences in demographic variables as they related to precautions taken during outdoor PA are demonstrated in Table 2. All demographic variables were found to significantly differ in terms of precautions taken during outdoor PA. Participants with an associate or college degree, with an annual income of US$30,001–70,000, who were male, who were married, who were African-American, who had a full-time job, who had at least one chronic disease, and who lived in an urban area had lower frequencies of taking precautions during outdoor PA. Participants’ age (*p* = 0.003) was positively correlated with the frequency of precautions taken during outdoor PA. Participants with a low perceived immune status had a significantly lower frequency of taking precautions during outdoor PA than those with a higher perceived immune status (*p* < 0.001).

**TABLE 2:** Differences of demographic variables and perceived immune status in terms of taking precautions during outdoor physical activity (United States, 2020).

Variable		Mean	SD	*F*	*p*	Post-hoc
Highest education	(1) Less than high school	2.52	0.56	4.58	**0.011**	2 < 3
	(2) Associate or college degree	2.43	0.54			
	(3) Graduate degree	2.65	0.50			
Annual income (US$)	(1) <30,000	2.50	0.55	5.06	**0.002**	2,3 < 4
	(2) 30,001–50,000	2.40	0.53			
	(3) 50,001–70,000	2.40	0.51			
	(4) >70,001	2.67	0.54			
Ethnicity	(1) White	2.56	0.56	8.84	**<0.001**	3 < 1
	(2) Asian	2.42	0.48			
	(3) African-American	2.19	0.42			
	(4) Other	2.48	0.53			
Marital status	(1) Single	2.57	0.55	13.74	**<0.001**	2 < 1<3
	(2) Married	2.39	0.52			
	(3) Other	2.87	0.43			
Employment status	(1) Full-time job	2.42	0.52	8.06	**<0.001**	1 < 3,4
	(2) Part-time job	2.55	0.59			
	(3) Unemployed	2.79	0.45			
	(4) Other	2.81	0.52			
Living environment	(1) Urban area	2.34	0.51	17.32	**<0.001**	1 < 2,3
	(2) Suburban area	2.63	0.55			
	(3) Rural area	2.58	0.49			
		Mean	SD	*t*	*p*	*d*
Gender	Male	2.39	0.52	−3.97	**<0.001**	0.377
	Female	2.59	0.54			
Chronic diseases	No	2.54	0.51	2.90	**0.004**	0.261
	Yes	2.40	0.56			
Perceived immune status	Low	2.10	0.38	−14.32	**<0.001**	1.329
	High	2.69	0.50			
				r^2^	*p*	
Age				0.15	**0.003**	

SD, standard deviation; d, Cohen’s d for effect sizes. The statistical significance level is set at 0.05 and the value of statistical significance is emphasized in bold.

### Perceived Immune Status, Health Beliefs, PA, and SB

Table 3 demonstrates the perceived immune status and health beliefs associated with the odds of having an active lifestyle or a sedentary lifestyle by a logistic regression model adjusted for covariables. Results revealed that the perceived immune status (odds ratio (OR) = 0.83 [95% confidence interval (CI) = 0.72–0.94], *p* = 0.005), perceived benefits of PA (OR = 3.16 [2.03–4.90], *p* < 0.001), and self-efficacy (OR = 3.03 [2.08–4.40], *p* < 0.001) were significantly associated with the odds of having an active lifestyle. Perceived barriers to PA, cues to action, susceptibility to disease, and social support did not reveal significance in the logistic regression model of an active lifestyle.

**TABLE 3:** Logistic regression model of active and sedentary lifestyles by perceived immune status and health beliefs (United States, 2020).

Dependent variable	Active lifestyle			Sedentary lifestyle		
Independent variable	OR	(95% CI)	*p*	OR	(95% CI)	*p*
Perceived immune status	0.83	(0.72–0.94)	**0.005**	1.06	(0.93–1.21)	0.387
Perceived barriers to PA	0.72	(0.45–1.14)	0.162	1.20	(0.75–1.92)	0.457
Perceived benefits of PA	3.16	(2.03–4.90)	**<0.001**	1.54	(0.96–2.47)	0.074
Cues to action	0.80	(0.53–1.23)	0.310	0.64	(0.41–1.00)	**0.048**
Susceptibility to disease	0.80	(0.57–1.13)	0.201	1.45	(1.02–2.06)	**0.038**
Social support	0.79	(0.54–1.16)	0.228	0.79	(0.54–1.15)	0.224
Self-efficacy	3.03	(2.08–4.40)	**<0.001**	0.66	(0.48–0.90)	**0.008**

PA, physical activity; OR, odds ratio; CI, confidence interval; Active lifestyle, adults who met physical activity recommendations. Sedentary lifestyle, sedentary time of ≥9 h/day; The logistic regression model was adjusted for continuous demographic variables. The statistical significance level is set at 0.05 and the value of statistical significance is emphasized in bold.

Regarding SB, results revealed that cues to action (OR = 0.64 [0.41–1.00], *p* = 0.048), susceptibility to disease (OR = 1.45 [1.02–2.06], *p* = 0.038), and self-efficacy (OR = 0.66 [0.48–0.90], *p* = 0.008) were significantly associated with the odds of having a sedentary lifestyle. The perceived immune status, perceived barriers and benefits of PA, and social support did not reveal significance in the logistic regression model of having a sedentary lifestyle.

### PA, SB, and QOL

Table 4 demonstrates differences in active and sedentary lifestyles in the four QOL domains. Participants with an active lifestyle had significantly higher scores in all QOL domains compared to those with an inactive lifestyle, including the physical (*p* = 0.024), psychological (*p* = 0.001), social (*p* < 0.001), and environmental domains (*p* < 0.001). Participants with a non-sedentary lifestyle had significantly higher scores of psychological (*p* = 0.049) and social (*p* < 0.001) domains of QOL compared to those with a sedentary lifestyle. Physical and environmental QOL did not reveal a significant difference in terms of SB.

**TABLE 4:** Difference in active and sedentary lifestyles in terms of the quality of life (QOL) (United States, 2020).

Group	Inactive lifestyle (*N* = 89)		Active lifestyle (*N* = 378)		*p*	d	Non-sedentary lifestyle (*N* = 397)		Sedentary lifestyle (*N* = 70)		*p*	d
QOL domain	Mean	SD	Mean	SD			Mean	SD	Mean	SD		
Physical health	14.43	3.19	15.26	2.59	**0.024**	0.286	15.08	2.64	15.63	3.05	0.134	0.193
Psychological health	13.34	3.89	14.78	2.90	**0.001**	0.419	14.70	3.04	13.74	3.54	**0.049**	0.291
Social relationships	13.15	4.35	15.07	3.56	**<0.001**	0.483	15.05	3.65	13.05	4.05	**<0.001**	0.519
Environment	14.17	3.10	15.88	2.73	**<0.001**	0.585	15.67	2.80	15.22	3.13	0.251	0.152

Active/Inactive lifestyles, adults who met/did not meet physical activity recommendations. Sedentary/Non-sedentary lifestyle, sedentary time of ≥9/<9 h/day; SD, standard deviation; d, Cohen’s d for the effect size. The statistical significance level is set at 0.05 and the value of statistical significance is emphasized in bold.

## Discussion

This study explored PA and SB in the early stage of the COVID-19 outbreak. Precautionary behaviors conducted when engaged in outdoor PA differed with all demographic variables. During the pandemic, one’s perceived immune status, benefits of PA, and self-efficacy were factors associated with an active lifestyle. Cues to action, susceptibility to disease, and self-efficacy were factors associated with individuals’ sedentary lifestyles. Participants with a non-sedentary lifestyle had better psychological and social QOL. Engaging in an active lifestyle was beneficial for promoting all domains of QOL during the COVID-19 pandemic.

In addition to home exercise, PA in the outdoor environment can bring additional health benefits, especially in terms of immune functions during a pandemic [7]. Individuals should continue being physically active at gyms, parks, and recreational facilities but should take necessary precautions to protect themselves [8]. Recommendations for precautions during outdoor PA should be followed before, during, and after PA sessions [15]. Based on the results of the current study, people who are less likely to take precautions during outdoor PA included those who are young, male, married, African-American, have a college degree, have an annual income of US$30,001–70,000, have a full-time job, have a chronic disease, live in an urban area, or have a low perceived immune status.

Regarding psychological factors associated with PA and SB, the current study found that one’s perceived immune status was a factor in whether one engaged in an active lifestyle. One’s perceived immune status was affected by stressors from the environment, psychology, and society related to COVID-19 events [26]. Individuals who may have been afraid of and worried about being infected by the virus had higher levels of susceptibility to COVID-19. These stresses can make vulnerable individuals feel uncomfortable and panic with negative thoughts which might lower their perceived immunity and discourage healthy behaviors [13, 14, 23, 27]. In a qualitative study, individuals indicated that during the outbreak, immunity was a concern responsible for downward trends in their PA, health status, self-care ability, social visits, QOL, and other health indicators [28].

This current study also applied the HBM in the logistic regression model of an active lifestyle and sedentary lifestyle. From a psychological perspective, individuals exhibited decreased motivation to engage in PA and increased opportunities for SB in the early stage of the pandemic [3, 28]. The current results showed that perceived benefits of PA were associated with an active lifestyle, while cues to action and susceptibility to disease were associated with a sedentary lifestyle. Individuals’ perceived benefits are an attitude toward PA which supports the intention with intrinsic rewards of healthy behaviors. Perceived benefits are positively associated with the intention to engage in and the habit of conducting healthy behaviors [11, 14]. Furthermore, self-efficacy is a strong and well-established psychological factor associated with PA and SB [29]. Similar to the findings of this study, self-efficacy seemed to be the most important determinant of PA and SB intentions [11, 30]. Overall, the HBM can be applied to explain psychological factors associated with PA and SB during a pandemic.

The current study revealed that the QOL of individuals who engaged in an active lifestyle and a non-sedentary lifestyle benefited during the pandemic, especially in the psychological and social relationship domains. Previous studies also suggested that PA and SB were linked to QOL in healthy populations. During the COVID-19 outbreak, PA was found to be significantly positively associated with QOL. SB was also found to be significantly negatively associated with QOL. Low PA and high screen time might be reasons for the reduced QOL during the pandemic [31]. Subjective PA was positively associated with the physical, psychological, and social domains of QOL [32]. Inactive participants had lower overall QOL scores compared to active participants [33]. Self-reported SB were significantly negatively associated with psychological QOL [32].

However, there are still several limitations to this study. We conducted a cross-sectional study *via* an internet survey on the Mturk platform. The sample might not be representative, because it only reached participants who can read English and have an active Amazon account. SB and PA were measured by self-reported questionnaires. The recall and subjective data could be biased in presenting the actual situation. This study only applied the perceived immune status and HBM as factors. There are still other factors that might affect PA and SB during a pandemic. Most importantly, rapid changes in the pandemic might have influenced the results of this study and its ability to infer future situations.

Based on the results and limitations of this study, suggestions were made for future research. The Mturk platform is an efficient approach for rapidly collecting data because it overcomes the difficulty of collecting data in person by face-to-face interviews during a pandemic [19]. The selection criteria should be pre-set in the Mturk platform with careful consideration of the study population to increase the sample representativeness [18]. Furthermore, SB and PA can be measured objectively to avoid bias [30]. Future studies can assess other potential factors (i.e., environment, policies) that could affect PA which might differ between pandemic and non-pandemic periods. Other theoretical models can be applied to explain changes in PA and SB behaviors during a pandemic, such as the Transtheoretical Model [34].

The study has practical implications for facing new and similar pandemics in the future. In the early stage of a pandemic outbreak, engaging in an active lifestyle and avoiding a sedentary lifestyle might be a good approach for promoting QOL [31]. Decreasing SB and increasing indoor and outdoor PA while taking necessary precautions would be beneficial during a pandemic [3, 5]. Health education about psychological factors affecting PA is a good practical health strategy [35], such as advancing self-efficacy, improving awareness of PA benefits, and explaining how to overcome PA barriers during a pandemic. Precautionary behaviors taken during outdoor PA should be advocated in times with or without a pandemic [15], especially in individuals with a low education, with a low income, with a full-time job, with chronic diseases, who are male, who are African-American, or who live in an urban area.

During a pandemic, increasing indoor and outdoor PA and decreasing SB are beneficial for achieving weekly PA recommendations. Individuals should be educated on precautionary behaviors and pay attention before, during, and after outdoor PA, especially those in vulnerable populations. Furthermore, strengthening health beliefs through health education might be an efficient strategy for improving PA and reducing SB during a pandemic. Individuals are recommended to engage in an active lifestyle and avoid a sedentary lifestyle when facing future unpredictable infectious pandemics similar to COVID-19, thereby enhancing their QOL.
